# Decreased NAD+ in dopaminergic neurons

**DOI:** 10.18632/aging.101433

**Published:** 2018-04-28

**Authors:** Samantha L. Sison, Allison D. Ebert

**Affiliations:** 1 Department of Cell Biology, Neurobiology and Anatomy, Medical College of Wisconsin, Milwaukee, WI 53226, USA

**Keywords:** Parkinson’s disease, LRRK2, sirtuins, mitochondra, iPSCs

Parkinson’s disease (PD) is the second most common age-related neurodegenerative disease, accounting for 1% of the population over 60 years of age [1]. PD is characterized by the loss of dopaminergic neurons in the substantia nigra which results in a variety of symptoms, most of which are related to motor function. One main question that remains unanswered in the field is what makes dopaminergic neurons so susceptible to disease. If we know what is going wrong within these cells, we may be able to create therapeutics to prevent degeneration. Factors that have been shown to be implicated in dopaminergic vulnerability include increased oxidative stress due to dopamine oxidation, higher metabolic activity, and calcium buffering defects, all of which may converge on mitochondrial malfunction.

In our recent work, we investigated mitochondrial function across multiple neuronal cell types derived from human induced pluripotent stem cells (iPSCs) carrying the G2019S mutation in leucine-rich repeat kinase 2 (LRRK2), a gene that has been associated with both familial and sporadic PD [2]. We observed a decrease in mitochondrial content and mitochondrial distribution in LRRK2 G2019S iPSC-derived dopaminergic neurons compared to control; however, LRRK2 G2019S iPSC-derived glutamatergic or sensory neurons were unaffected. In addition, we observed an increase in the velocity of mitochondria moving toward the cell body only in the LRRK2 G2019S iPSC-derived dopaminergic neurons, correlating with the decreased number of mitochondria in the distal neurite. Moreover, we found altered mitochondrial respiration and ADP and ATP levels in LRRK2 G2019S iPSC-derived dopaminergic neurons compared to control. Together, these data suggest that LRRK2 G2019S expressing dopaminergic neurons exhibit certain intrinsic mitochondrial defects compared to other LRRK2 G2019S expressing neurons. We next wanted to further investigate possible cellular mechanisms that may be underlying these phenotypes. One cellular regulation system that has increasingly been implicated in proper mitochondrial function is protein acetylation [3]. Thus, we examined the expression of sirtuins, which are NAD+ dependent protein deacetylases that have been found to play anti-aging, anti-oxidant, and metabolic roles within the nucleus, cytosol, and mitochondria [4]. We hypothesized that sirtuin levels would be decreased considering the dramatic mitochondrial deficits observed. Unexpectedly however, three sirtuins, sirtuin (SIRT) 1, SIRT2, and SIRT3, were upregulated in the LRRK2 G2019S iPSC-derived dopaminergic neurons compared to controls [2]. However, acetylation of specific sirtuin targets were increased in the LRRK2 G2019S iPSC-derived dopaminergic neurons, suggesting that the activity of these deacetylases was diminished. Neither LRRK2 G2019S iPSC-derived glutamatergic neurons nor sensory neurons had altered sirtuin expression or deacetylation function. Since sirtuins require NAD+ to function, we asked whether NAD+ levels were altered in the LRRK2 G2019S iPSC-derived dopaminergic neurons, and indeed that was what we observed. Interestingly, NAD+ levels were significantly reduced in both control and LRRK2 G2019S iPSC-derived dopaminergic neurons compared to glutamatergic and sensory neurons, but NAD+ levels were more dramatically decreased in the LRRK2 G2019S dopaminergic neurons compared with controls. Taken together, we have identified a misregulation in sirtuin activity in human PD cells that likely is due to decreased NAD+ levels, which is seemingly specific to dopaminergic neurons.

NAD+ is a key regulator of many important cellular processes including oxidative metabolism mediated by sirtuins. With low levels of NAD+ in the LRRK2 G2019S iPSC-derived dopaminergic neurons, increased levels of acetylation of known sirtuin targets, and robust mitochondrial defects, an important question arises: can NAD+ be used as a therapeutic target to help prevent dopaminergic degeneration in PD? Not surprisingly, there are many studies providing evidence for using NAD+ as a therapeutic target for a variety of applications from general aging to neurodegenerative disorders [5]. However, the use of NAD+ boosting therapeutics and NAD+ precursors in PD is not well elucidated. A small number of studies have tested NAD+ precursors or NAD+ boosters in fly and rodent PD models, and most of the reports suggest that increasing NAD+ levels are beneficial. For example, one study found a decrease in oxidative stress and an increase in mitochondrial function in a Drosophila model of PD when diets were supplement with high doses of nicotinamide [6]. However, a clinical case report documented adverse side effects after treating a PD patient with the NAD+ precursor niacin, despite an improvement in motor function [7]. Much more research is needed to test the use and efficacy of NAD+ precursors and activators so that any potential benefits for dopaminergic neurons do not come at the expense of the health of other cell types. Nevertheless, the hope is that when we learn more about the NAD+ requirements within dopaminergic neurons, we will better understand PD pathogenesis, which will help us develop therapeutics that prevent the dopaminergic loss in PD.

## References

[1] Reeve A, et al. Ageing Res Rev. 2014; 14:19–30. 10.1016/j.arr.2014.01.004 24503004 PMC3989046

[2] Schwab AJ, et al. Stem Cell Reports. 2017; 9:1839–52. 10.1016/j.stemcr.2017.10.010 29129681 PMC5785678

[3] Baeza J, et al. Trends Biochem Sci. 2016; 41:231–44. 10.1016/j.tibs.2015.12.006 26822488 PMC4783225

[4] Herskovits AZ, Guarente L. Cell Res. 2013; 23:746–58. 10.1038/cr.2013.70 23689277 PMC3674397

[5] Rajman L, et al. Cell Metab. 2018; 27:529–47. 10.1016/j.cmet.2018.02.011 29514064 PMC6342515

[6] Jia H, et al. J Neurosci Res. 2008; 86:2083–90. 10.1002/jnr.21650 18381761

[7] Alisky JM. Nutr Neurosci. 2005; 8:327–29. 10.1080/10284150500484638 16669604

